# A Novel Technique for the Evaluation and Interpretation of Elastography in Salivary Gland Involvement in Primary Sjögren Syndrome

**DOI:** 10.3389/fmed.2022.913589

**Published:** 2022-05-31

**Authors:** Rosa Elda Barbosa-Cobos, Rubén Torres-González, Ana Victoria Meza-Sánchez, Lucio Ventura-Ríos, Luz Elena Concha-Del-Río, Julián Ramírez-Bello, Everardo Álvarez-Hernández, Claudia Irene Meléndez-Mercado, Favio Edmundo Enríquez-Sosa, Cinthia Jahoska Samuria-Flores, Gustavo Esteban Lugo-Zamudio, Cristina Hernández-Díaz

**Affiliations:** ^1^Servicio de Reumatología, Hospital Juárez de México, Centro Médico ABC y Grupo “Manifestaciones Oculares en Reumatología” MOR, Mexico City, Mexico; ^2^Dirección de Educación e Investigación en Salud, Unidad Médica de Alta Especialidad (UMAE) de Traumatología, Ortopedia, Rehabilitación “Dr. Victorio de la Fuente Narváez,” Instituto Mexicano del Seguro Social (IMSS) y Grupo “Manifestaciones Oculares en Reumatología” MOR, Mexico City, Mexico; ^3^Escuela Superior de Medicina, Instituto Politécnico Nacional, Mexico City, Mexico; ^4^División de Reumatología, Instituto Nacional de Rehabilitación “Luis Guillermo Ibarra Ibarra,” Mexico City, Mexico; ^5^Clínica de Enfermedades Inflamatorias Oculares, Asociación Para Evitar la Ceguera en México (APEC), Hospital de la Ceguera “Dr. Luis Sánchez Bulnes,” y Grupo “Manifestaciones Oculares en Reumatología” MOR, Mexico City, Mexico; ^6^Departamento de Endocrinología, Instituto Nacional de Cardiología, Mexico City, Mexico; ^7^Servicio de Reumatología, Hospital General de México, “Dr. Eduardo Liceaga,” Mexico City, Mexico; ^8^Servicio de Reumatología, Centro Médico ISSEMYM “Lic. Arturo Montiel Rojas” Toluca, Mexico City, Mexico; ^9^Servicio de Reumatología, Hospital Regional “General Ignacio Zaragoza,” ISSSTE, Mexico City, Mexico

**Keywords:** major salivary glands, ultrasound, elastography, pixel analysis, primary Sjögren syndrome

## Abstract

Ultrasound (US) of major salivary glands (MSG) evaluates echogenicity, border features and vascularization, with elastography, it can detect tissue elasticity and glandular fibrosis, related to inflammation in Primary Sjögren’s syndrome (pSS). This study aimed to develop a novel technique by pixel analysis for evaluation and interpretation of elastography in MSG in pSS. A cross-sectional and observational multicenter study was conducted. The US of MSG performed in orthogonal planes in grayscale, Doppler, and shear-wave elastography. For elastography images of each gland were analyzed with the open-source program ImageJ to perform a pixel analysis. Statistical analysis was performed with the IBM-SPSS v25 program. Fifty-nine women with a mean age of 57.69 (23–83) years were recruited; pSS mean duration of 87 (5–275) months, and 12 healthy women without sicca symptoms as a control group with a mean age of 50.67 (42–60) years. Intragroup analysis showed *p*-values >0.05 between sicca symptoms, ocular/dryness tests, biopsy, US, and pixel analysis; correlation between Hocevar and pixel analysis was not found (rho < 0.1, *p* >0.5). MSG anatomical size was 41.7 ± 28.2 mm vs. 67.6 ± 8.8 mm (*p* ≤ 0.0001); unstimulated whole saliva flow rate was 0.80 ± 0.80 ml/5 min vs. 1.85 ± 1.27 ml/5 min (*p* = 0.016). The elastography values (absolute number of pixels) were 572.38 ± 99.21 vs. 539.69 ± 93.12 (*p* = 0.290). A cut-off point risk for pSS identified with less than 54% of red pixels in the global MSG mass [OR of 3.8 95% CI (1.01–15.00)]. Pixel analysis is a new tool that could lead to a better understanding of the MSG chronic inflammatory process in pSS.

## Introduction

Primary Sjögren’s syndrome (pSS) is a chronic, systemic, and autoimmune exocrinopathy involving mainly the salivary and lacrimal glands, which are progressive destructed by an immune-mediated process. Xerostomia and xeropthalmia are the main complaints, but other sicca symptoms and extra-glandular manifestations may be present (1). pSS confirmation of depends on objective measures of dysfunctional salivary or lacrimal glands in addition to serological or salivary gland histopathology of MSG to evidence autoimmunity. Salivary gland correct evaluation of involvement provides data for the pSS diagnosis (2, 3).

The major salivary glands (MSG) are the parotid, submandibular and sublingual. Meanwhile, the minor salivary glands are located throughout the mouth and the aerodigestive tract.

The glands, especially parotid, are also composed of abundant fatty tissue with a ratio of adipose-acinar tissue 1:1 (4). All salivary glands can be affected by pSS (4, 5). The parotid and submandibular glands, if affected by inflammation, contribute little to saliva production. Acini atrophy can derive from ductal system failure and influence xerostomia. In the beginning, the affection in pSS is related to peripheral intraglandular ducts and acini, due to inflammation associated with lymphocytic infiltrate (foci) located around the ducts, with the capacity to carry out effective immune responses (5).

Although imaging techniques contribute to the diagnosis by avoiding invasive procedures, such as biopsies, there is no gold standard technique to evaluate MSG. Due to its accessible location, it is easier to determine structural damage. Magnetic resonance imaging (MRI) has proven good performance, with a specificity of up to 98%, but it is expensive, whereas scintigraphy and sialography with specificities of up to 50 and 82%, respectively, are not widely used due to their invasiveness and high cost (6, 7). Ultrasound (US) has proven good sensitivity and very good specificity when compared to sublingual biopsies, using different scoring systems, equipment, and transducers (8, 9). US is non-invasive, non-expensive, non-ionizing radiation-related, and easy-going for patients. MSG US evaluates echogenicity, border features in B mode, and vascularization with power Doppler, with high specificity (between 83 and 98%) (9). Today, MSG US is used to describe glandular homogeneity, echogenicity, and parenchymal characterization, with good performance when compared to labial biopsies, minor salivary gland focus score and other autoimmune diseases (10), and provides accurate information about intraglandular vascularization.

Recently, elastography has been used to detect tissue elasticity and glandular fibrosis (11–13); there is scarce information in elderly pSS patients in which MSG US is associated with glandular atrophy (14). There is no valid scoring system to interpret elastography in MSG, however, a four-grade score has been used, which gives an interpretation related to the main color in the area of interest. To achieve better objective evaluation, with qualitative elastography, some image visualization software has been used, one of them, Image J (NIH^®^) (15), allows delimiting the area of interest by obtaining a histogram of the color map, with which you can get obtained a quantitative analysis of the pixels in general and each color.

However, there is still the need for a better approach to evaluate MSG involvement in daily clinical settings that could be easily applied and learned.

This study aimed to develop a novel technique by pixel analysis for evaluation and interpretation of elastography in MSG in pSS.

## Patients and Methods

### Subjects and Study Design

A cross-sectional and observational multicenter study was conducted. The study was done according to the ethics guidelines of the Declaration of Helsinki and approved by the ethics, research, and biosecurity Committees of Hospital Juárez de México (HJM0323/17-R) and Instituto Nacional de Rehabilitación “Luis Guillermo Ibarra Ibarra” (INRLGII 01/21/SP-1). All participants signed a written informed consent letter.

### Clinical Assessment

Epidemiological, clinical, serological, histological, and therapeutic data were abstracted from clinical records. Patients with pSS (who fulfilled the 2016 American College of Rheumatology-European League Against Rheumatism classification criteria for Sjögren syndrome) enrolled consecutively, and the control group was integrated by healthy women (without sicca symptoms). All subjects were examined to evaluate sicca symptoms, clinical parotid gland enlargement, systemic manifestations, and ocular and oral dryness, last assessed with unstimulated whole saliva flow rate for salivary flow measurement according to Navazesh et al. (16). Labial salivary gland biopsy was only performed in pSS group for ethical issues. The exclusion criteria were the coexistence of another autoimmune disease.

### Major Salivary Glands Ultrasound and Elastography

After clinical evaluation, all participants were sent to the US unit.

US and elastography were performed by the same ultrasonographer (HDC) with 18 years of experience, blinded to subject data in a mean time of 15 min.

Grayscale and elastography from parotid and submandibular gland were assessed, with subject in sitting position and the neck slightly extended backward, in orthogonal planes bilaterally. A GE Logic P7 unit was used with a 6–12 MHz multifrequency linear transducer for grayscale and a 10 MHz frequency power Doppler with PRF 0.6; color gain was adjusted when sound artifacts disappeared below cortical bone. Shear wave elastography of each gland took in the same position as the grayscale, displaying images simultaneously, showing the region of interest (ROI) when the compressibility limit come to green in the visual indicator scale on the screen. The softest component was described in red, whereas the hardest was depicted in blue, and green showed intermediate elasticity. A total of thirty-six images of each subject were saved and subsequently evaluated to determine the grayscale MSG structural changes using the Hocevar scoring system (0–48, cut-off score 18 points) and the presence of Doppler signal excluding normal vessels (17). The open-source program ImageJ was used for image pixel analysis, both in greyscale and elastography.

### Statistical Analysis

A specific integrated database was used for our study with Excel and IBM-SPSS v25. Description of variables made with measures of central tendency and dispersion. Homogeneity variable analysis with Chi-square for nominal and Levene’s statistic for numerical carried out, *p*-values > 0.05. Variables compared between groups (pSS patients and healthy subjects) and intragroup (pSS patients) with chi-square and means with Student’s *t*-test, ANOVA, Pearson and Spearman correlation; with statistical significance *p*-values ≤ 0.05, with the calculation of impact measure through risks with OR, and identification of cut-off point with statistical significance through CI at 95%.

## Results

The study group 59 women with a mean age of 57.69 (23–83) years, mean disease duration of 87 (5–275) months, and the control group was 12 healthy women with a mean age of 50.67 (42–60) years.

Clinical, serological, and histological characteristics of pSS group are depicted in Table 1. No clinical parotidomegaly was found among the patients or healthy subjects. Systemic treatment of the patients consisted in hydroxychloroquine 30 (50.8%), glucocorticoids 22 (37.3%), methotrexate 20 (33.9%), rituximab 3 (5.1%), cyclophosphamide 1 (1.7%), and mycophenolate 1 (1.75%).

**TABLE 1:** Characteristics and treatment of 59 women with primary Sjögren syndrome.

Variable	Frequency	(%)
**Sicca symptoms**		
Xeropthalmia	59	100
Xerostomia	42	71
Nasal dryness	16	27
Non-productive cough	5	8.5
Vaginal dryness	11	18.6
Cutaneous dryness	17	28.8
**Ocular dryness tests**		
Positive schirmer-1 test^#^	30	50.8
Ocular staining score^&^	33	55.9
Tear break-up time*	56	94.9
**Oral dryness tests**		
Unstimulated whole saliva flow rate^$^	35	59.3
**Systemic manifestations**		
Fatigue	14	23.7
Fever	1	1.7
Night sweats	13	22
Involuntary weight loss	5	8.5
Arthralgias	13	22
Synovitis	5	8.5
Raynaud phenomenon	3	5.1
Anti-SSA (Ro)^+^	51	86
Anti-SSB (La)^+^	31	52.5
**Labial salivary gland biopsy^‡^**		
Positive	50	84.7
Negative	3	5.3
Not performed	6	10
**Symptomatic treatment**		
Ocular	58	98
Oral	17	28.8
Vaginal	13	22
Cutaneous	38	64
**Comorbidity**		
Smoking	8	13.6
Diabetes	2	3.4
Hypertension	9	15.3
Dyslipidemia	16	27.1
Hypothyroidism	14	23.7

^#^≤5 mm/5 min on at least one eye, ^&^≥ 5 on at least one eye, *<10 s, ^$^≤ 0.1 ml/min, ^‡^with focal lymphocytic sialadenitis and focus score ≥ 1 foci/4 mm^2^. +, Positive.

Intragroup analysis showed *p*-values > 0.05 between sicca symptoms, ocular and dryness tests, systemic manifestations, labial salivary gland biopsy, US glandular size, and pixel analysis. Correlation between Hocevar and pixel analysis was not found (rho < 0.1, *p* > 0.5).

**FIGURE 1:**
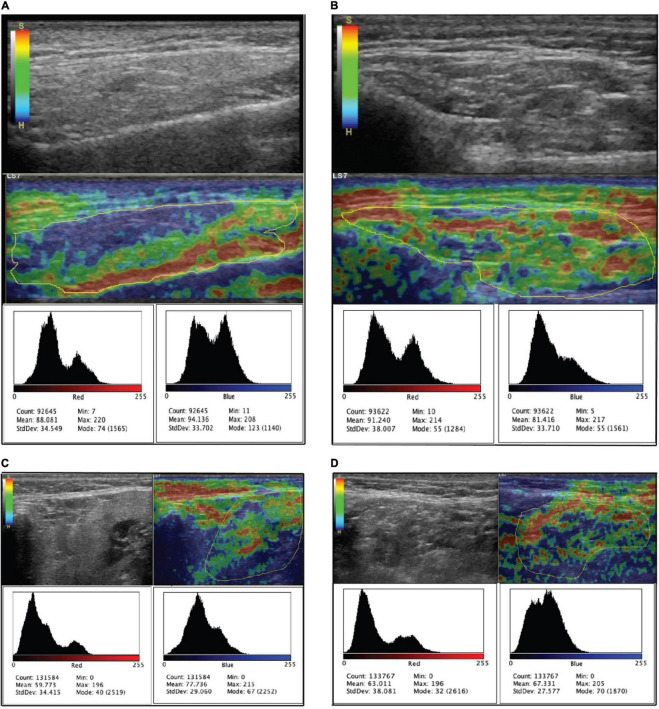
Shows the B-mode ultrasound and elastography of major salivary glands. Below each ultrasound figure are the histograms for soft (red) and hard (blue) tissues. **(A,C)** Are the parotid and submandibular glands of a patient with a Hocevar score of 1, and **(B,D)** are the glands of a patient with a Hocevar score of 33.

The anatomical size of all MSG identified were 41.7 ± 28.2 mm for Sjögren patients and 67.6 ± 8 mm for healthy subjects (group 1: 38.8% smaller), with a *p*-value < 0.0001; unstimulated whole saliva flow rate was 0.80 ± 0.80 ml/5 min for group 1 and 1.85 ± 1.27 ml/5 min for group 2, with a *p*-value of 0.016. The elastography values by an absolute number of pixels were 572.38 ± 99.21 in group 1 and 539.69 ± 93.12 in group 2 (*p*-value of 0.290). A cut-off point risk was identified for pSS with less than 54% of Red Pixels in the global salivary gland mass, with OR of 3.8 95% CI (1.01–15.00) (Figure 1).

When comparing elastography values between groups, with and without sicca symptoms, they showed *p*-values > 0.005.

## Discussion

We explored a new face of US using elastography to evaluate pSS. US and elastography gave us a new non-invasive approach to possibly integrate these features into pSS diagnosis.

By using pixel analysis, we demonstrated that global salivary gland mass was < 54% red without a predominant stiffness, as also shown by Zhang et al., which used a global score derived from the one used for hepatic fibrosis with chronic hepatitis C (18, 19). In this study, Zhang et al. also demonstrated that elastography scores of bilateral parotids correlated significantly with dental loss and disease duration.

In our study, we found by elastography stiffer tissue in both parotid and submandibular glands by pixel analysis, the finding was associated significantly with minor glands and low unstimulated whole saliva flow rate; it can translate as chronic damage or its consequences; differing to what Zhang et al. found in their study in which the parotid was the most involved gland (20, 21).

This finding could be related to fatty tissue infiltration in the glands. Additionally, it accompanies the initial inflammatory and fibrotic process, which is not evaluated adequately since parotid or submandibular biopsies are not recommended (22). However, fatty tissue is an inflammatory tissue, so detecting initial changes with elastography, especially with pixel analysis leads to early diagnosis of inflammation or fibrosis and fatty infiltrate in the gland; as has been done in other studies in which elasticity index has been used (13, 20), but not this novel pixel analysis technique. Our finding suggests a path, possibly related to fatty tissue inflammation, to evaluate early damage, as Skarstein et al. (22) previously showed. They performed a biopsy of labial salivary glands, finding that the fatty tissue that replaced glandular tissue was rich in interleukin 6, especially in areas in which adipocytes were about focal infiltrates. This hypothesis should be further studied for MSG without invasive procedures, so elastography is a good possibility.

Jimenez-Royo et al. (23) have demonstrated by MRI that there is a fatty infiltrate in the MSG, possibly resembling functional glandular tissue substitution, even though salivary flow rate has a weak correlation with the imaging technique, as in our study.

Despite the small number of patients, we found that pixel analysis gives accurate information related to MSG structural glandular changes.

US allows us to explore new tissue characteristics such as elasticity, picturing new ways to analyze MSG structural damage, by exploring the fat deposit role during pSS natural history.

## Conclusion

Even though in the pSS group, mass in the MSG is 38.3% smaller, the global number of pixels is similar in both groups, contrasting with the lower saliva production and less elastic tissue; that is shown by the lower proportion of red pixels in the pSS group. The identified cut-off point could function as a new screening test in this group of patients.

Pixel analysis is a new tool that could lead to a better understanding of the chronic inflammatory process in pSS by showing the role that fat tissue deposition plays in the fibrotic process of the major salivary glands.

## Data Availability Statement

The raw data supporting the conclusions of this article will be made available by the authors, without undue reservation.

## Ethics Statement

The studies involving human participants were reviewed and approved by the Hospital Juárez de México (HJM0323/17-R) and Instituto Nacional de Rehabilitación “Luis Guillermo Ibarra Ibarra” (INRLGII 01/21/SP-1). The patients/participants provided their written informed consent to participate in this study.

## Author Contributions

RB-C, RT-G, AM-S, LV-R, and CH-D: manuscript elaboration, conception and edition, statistical analysis, and final review. RB-C and RT-G: patient recruitment and data-based review. LC-D-R, JR-B, EÁ-H, CM-M, FE-S, CS-F, and RB-C: patient recruitment. RB-C, RT-G, LV-R, and CH-D: final draft review and edition. All authors contributed to the article and approved the submitted version.

## Conflict of Interest

The authors declare that the research was conducted in the absence of any commercial or financial relationships that could be construed as a potential conflict of interest.

## Publisher’s Note

All claims expressed in this article are solely those of the authors and do not necessarily represent those of their affiliated organizations, or those of the publisher, the editors and the reviewers. Any product that may be evaluated in this article, or claim that may be made by its manufacturer, is not guaranteed or endorsed by the publisher.
